# A study on the influence of dominant sound sources on users’ emotional perception in a pediatric dentistry clinic

**DOI:** 10.3389/fpsyg.2024.1379450

**Published:** 2024-05-23

**Authors:** Yang Liu, Xi Chen

**Affiliations:** ^1^School of Art and Design, Zhejiang Sci-Tech University, Hangzhou, China; ^2^College of Civil and Architectural Engineering, Heilongjiang Institute of Technology, Harbin, China

**Keywords:** pediatric dentistry clinic, soundscape, dominant sound source, emotional perception, healthcare workers, young pediatric patients

## Abstract

**Introduction:**

Soundscape in dental clinics has varying degrees of impact on the emotions of healthcare workers and young patients. Emotions such as restlessness, anxiety, anger, and nervousness are commonly found among dental healthcare workers. Pediatric dental clinics are an important part of dental clinics, but there is a lack of research on the soundscape within pediatric dental clinics.

**Methods:**

This study focuses on a typical pediatric dental clinic, using a combination of field questionnaires and objective measurements. It aims to determine the impact of dominant sound sources on the emotional perception (nervousness, restlessness, anger, fear, pain) and hostile emotional responses of users in the pediatric dental clinic.

**Results:**

In the soundscape of pediatric dental clinics for young pediatric patients, users experience negative emotional perceptions (nervousness, restlessness, anxiety, anger, fear, and pain) and emotional responses of hostility. The dominant sound sources can be divided into two categories: dental (dental drill, air-water syringe, and saliva ejector) and nondental (children crying). Under the influence of dental dominant sound sources, there was a significant negative correlation between the emotional perceptions of healthcare workers and their negative emotional perceptions (*p*  <  0.05). Conversely, for young pediatric patients aged 0–11  years, a significant positive correlation was observed between their emotional perceptions and negative emotional perceptions. The mean perceived degrees of nervousness and fear in young pediatric patients were 1.82 and 1.71 times stronger, respectively, than those observed in healthcare workers. Under the influence of non-dental dominant sound sources, the average degree of emotional perception among healthcare workers was 0.71 higher than that of young pediatric patients, and anxiety perception was significantly enhanced (*p*  <  0.05). The mean degree of nervousness perception was 1 point higher in healthcare workers compared to young pediatric patients, restlessness perception was 1.1 stronger, and there was a presence of mild pain perception. In terms of demographic/social factors, age, occupation, and years of work significantly affected the perceptions of fear and restlessness among healthcare workers, while age had a significant impact on the emotional reaction of hostility in young pediatric patients.

**Discussion:**

The results of this study indicate that the soundscape is an important factor in creating a comfortable treatment environment in pediatric dental clinics. Healthcare workers and young pediatric patients are significantly affected by the dominant sound sources in the clinic, and these effects are closely related to demographic and social factors such as age, profession, and years of experience. This finding can provide more targeted methods and strategies for the design and creation of soundscapes in dental clinics.

## 1 Introduction

A soundscape is an acoustic environment perceived by an individual, group, or community within a given scene (ISO, 2014). Sound comfort is an important indicator for the evaluation of soundscape quality. Axelsson et al. (2010) constructed a soundscape quality prediction model based on dimensions of pleasure, eventfulness, and familiarity to facilitate the measurement and improvement of soundscape quality; sound comfort is influenced not only by the background sound pressure level within the sound scene but also by the individual sound sources within the background noise (Yang and Kang, 2005, 2013; Kang et al., 2012); sound comfort also depends on an individual’s psychological state (Lorenzino et al., 2020), with emotional perception being a significant influencing factor of the psychological state (Herranz-Pascual et al., 2019). Kong and Han (2024) have found significant correlations between individuals’ psychological and physiological responses within the soundscape through a systematic review.

The study of soundscape evaluation from an emotional dimension has become an important direction today. Existing research has found that surveys created from the emotional dimension of sound can clearly and reliably reflect users’ positive and negative emotional perceptions within a soundscape (Masullo et al., 2021). In urban public open space soundscape research, Cain et al. (2013) used principal component analysis to categorize the emotional dimensions describing urban soundscapes into two types: calmness and vibrancy. From the perspective of sound sources, the sound sources in different urban scenes significantly affect people’s emotional perception, users would have a positive emotional response to the sound source they focus on (Jo and Jeon, 2021). Therefore, appropriately increasing people’s particular types of activities can enhance their sense of relaxation (Jo and Jeon, 2021; Zhang and Kang, 2022). Research on emotion perception in sound sequences indicates that users’ negative emotions can be modulated by the placement of different sound sources in the foreground and background noise with appropriate settings. In studies on the perception of dominant sound sources on emotion perception, it has been found that the acceptability of dominant sounds is consistent with people’s primary emotional perception (Xu et al., 2019). The perception of dominant sound sources on emotions such as nervousness, comfort, and pleasure perception has been identified. Studies in the activity spaces of elderly care facilities have identified the effects of dominant sound source types on elderly people’s feelings of pleasure, arousal, and annoyance (Qin et al., 2020). Research on the emotional experience of sound demonstrates that sound has immense potential to evoke emotional experiences. Emotional experience differs from complex emotions (such as depression and anger) and basic sensations (like/dislike) in that it includes two dimensions: valence and arousal. Valence refers to a subjective sensation of pleasure or displeasure, defining the hedonic value of an experience; arousal refers to a subjective state of being activated or deactivated, and defines the intensity of an experience (Russell, 1980, 2003; Özcan, 2014). Everyday experiences of sounds like dental drills and shavers indicate, from a phenomenological standpoint, that these sounds are ‘annoying, obtrusive, and irritating’ (Özcan and van Egmond, 2012). These emotional experiences fall on the negative side of the valence dimension, providing evidence of unpleasant experiences (Özcan, 2014), and these sounds also evoke negative emotional perceptions and reactions during human interactions.

The acoustic environment in a dental clinic may damage the hearing of healthcare workers and affect their emotional perception. Studies have shown that long-term exposure to noise of different frequencies and with sound pressure levels higher than 80 dB will cause hearing impairment in 40% of dentists (Akbakhanzadeh, 1978; Altinöz et al., 2001; Hyson, 2002; Gijbels et al., 2006; Bali et al., 2007; Mojarad et al., 2009; Fernandes et al., 2012). Moreover, several studies on the noise levels of medical devices have shown that the sound pressure levels of dental devices (e.g., high-speed turbine handpieces, dental tools, ultrasonic equipment) are within 85 dB during normal operation, but the noise levels of older devices are outside this range (Jadid et al., 2011; Kadanakuppe et al., 2011; Messano and Petti, 2012; Singh et al., 2012). The American Dental Association confirmed that dental instrument noise can cause hearing loss in healthcare workers through regular assessments of dentists’ hearing (Lopes et al., 2012). The sound environment in the dental clinic has different degrees of influence on the emotions of healthcare workers (Jones and Davies, 1984; Kjellberg et al., 1996); dental noise impacts the normal tasks of healthcare workers (Lopes et al., 2012), and restlessness, anxiety, anger and nervousness are commonly experienced among dental healthcare workers (Babisch, 2003; Chopra and Pandey, 2007; Wong et al., 2011, 2015; Chen et al., 2013; Muppa et al., 2013; Porritt et al., 2013). The pediatric dental clinic is an integral part of dental clinics. However, few studies have been conducted on the soundscape in these clinics, and there is a lack of studies examining the impact of dominant sound sources on the emotional perception of users within pediatric dental clinics.

Therefore, this article takes a typical pediatric dental clinic as the study subject and utilizes a combination of onsite surveys and objective measurements as research methods. The goal is to determine the effects of dominant sound sources on various emotional perceptions (nervousness, irritability, anger, fear, and distress) and emotional responses (hostility) of users within the pediatric dental clinic. Firstly, it was determined that emotional perception is a factor influencing users’ evaluation of the soundscape in pediatric dental clinic. Then, considering the identification and classification of sound sources, the dominant sound sources affecting users’ emotional changes were identified. Next, the patients were divided into two categories, i.e., healthcare workers and young pediatric patients, and the sound sources that significantly induced different emotional perception among users were identified as the dominant sound sources. Finally, the influence of these dominant sound sources on users’ emotional perception was determined according to demographic and social factors.

## 2 Methods

Considering the typicality and representativeness of this study, this paper adopted the pediatric dental clinic of the Department of Dentistry at the First Hospital of Harbin Medical University as the research site for collecting samples. The First Hospital of Harbin Medical University is a comprehensive tertiary hospital that integrates medical treatment, teaching and research and is the largest medical center in Heilongjiang province.

The field survey of the soundscape and emotional perception in the pediatric dentistry clinic was divided into two parts, i.e., interviews and field questionnaires. According to the concept of acoustic biotopes (Özcan et al., 2022), a dental clinic is viewed as a biotope, with species including healthcare workers and young pediatric patients. Therefore, based on different audience groups, respondents in the pediatric dental clinic were categorized into two groups for the interviews conducted in Chinese: healthcare workers and young pediatric patients; young pediatric patients were interviewed and surveyed with the accompaniment of their family members. First, in a pediatric dental clinic, 6 healthcare workers and 6 young pediatric patients were randomly selected for semi-structured interviews. The age range of the pediatric patient sample in this study was controlled to be between 0 and 11 years old (the interview outline is shown in Table 1). What people hear depends on who is listening, and the way people listen can affect the likelihood of interacting with different components of the environment (Özcan et al., 2022). Therefore, based on the results of the interviews, the pediatric dental clinic includes 10 types of sound sources. By extracting the sound sources unique to the dental clinic and the statements from two types of subjects about the relationship between sound sources and the emotions they evoke during the interviews, the sound sources are categorized into two types: dental and non-dental. The dental sound sources include the sound of a dental drill, a saliva ejector and an air/water syringe. Non-dental sound sources included conversations, children crying, parents crying/scolding, nurse calling patients’ name, air conditioning, and mobile ringtones. In the interviews, the emotional descriptors mentioned by respondents can be divided into two categories: positive emotions (pleasure and relaxation) and negative emotions (nervousness, restlessness, anxiety, anger, fear, pain, and hostility). In the questionnaire, adjectives identifying these two types of emotions are used to enable respondents to quickly and accurately determine their emotional perception in the soundscape of pediatric dental clinics.

**Table 1:** Semi-structured interview outline.

Interview content
1. What sounds can be heard in the dental clinic?
2. What are the sounds that impress you the most?
3. What are the sounds that disturb you the most?
4. What impact did these sounds have on your emotions?

**Figure 1:**
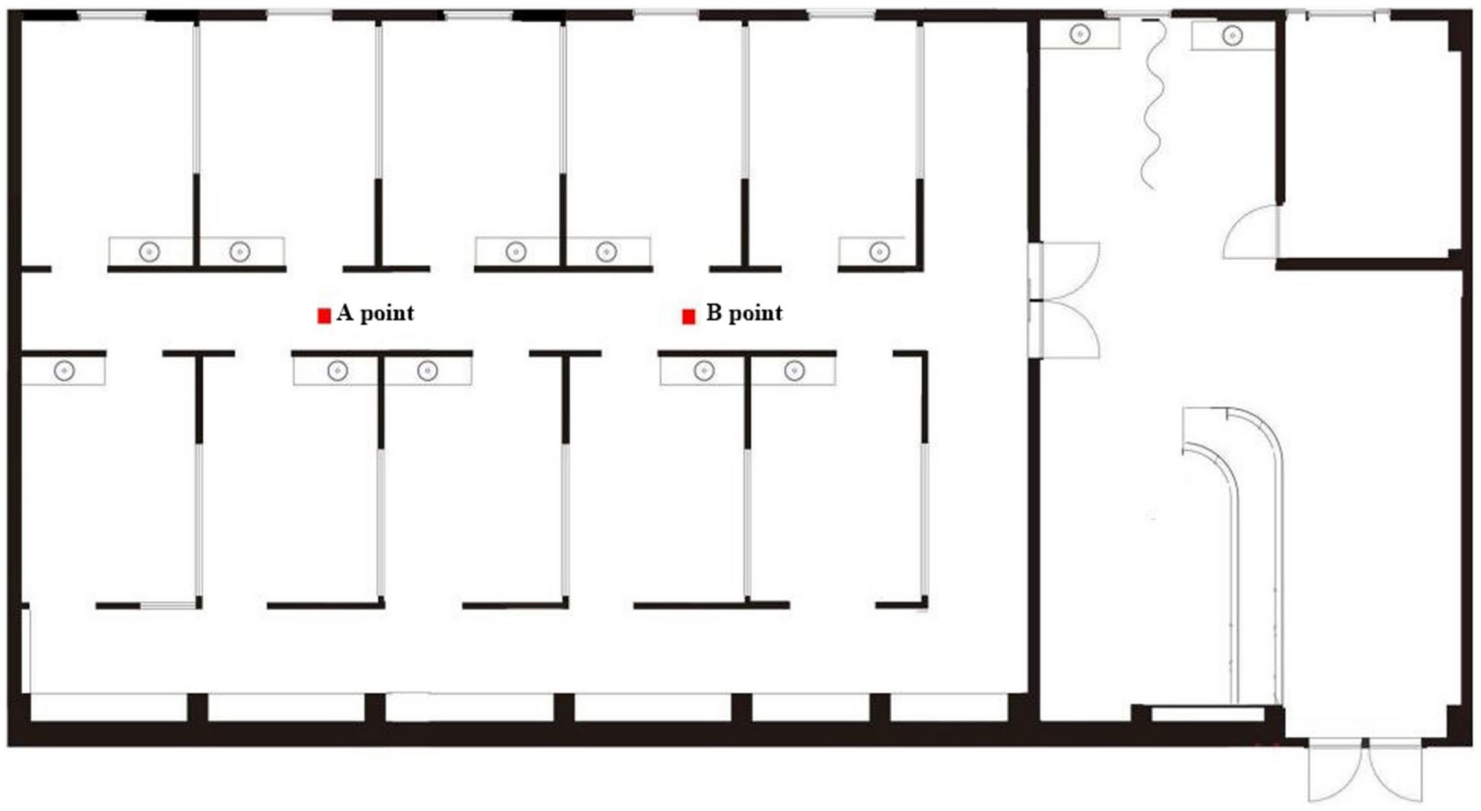
Sound pressure level measurement point map inside the pediatric dentistry clinic.

**Figure 2:**
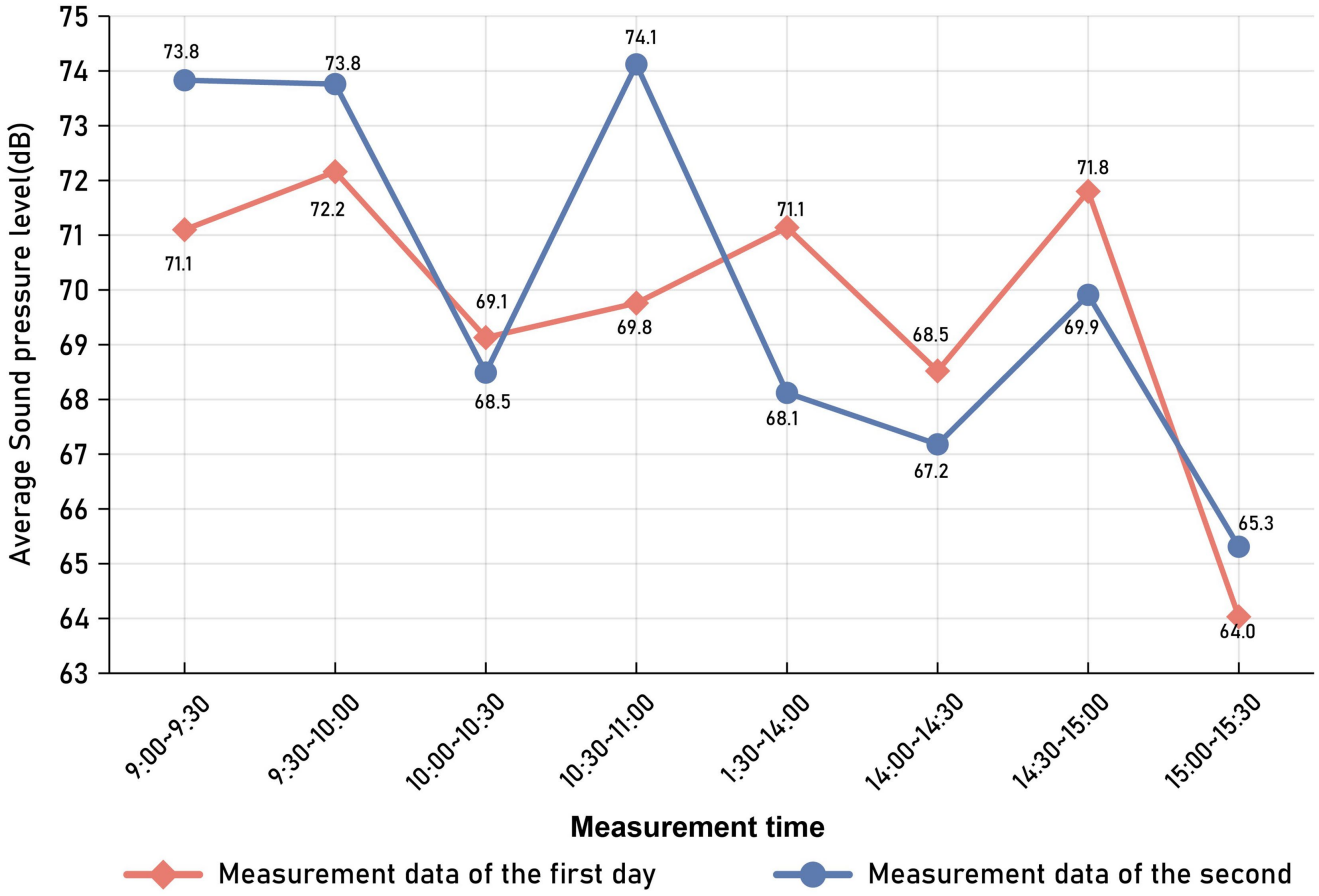
Curve of sound pressure level changes over time in the pediatric dental clinic.

The field questionnaire survey was conducted in January 2023. Initially, this study utilized field measurements to fully understand the variation patterns of the sound field inside pediatric dental clinics. During the working days of the clinic (Monday to Friday), two consecutive days (Wednesday and Thursday) were randomly selected for full-period testing during working hours, with testing times from 9:00 AM to 11:00 AM and from 1:30 PM to 3:30 PM. By testing the sound pressure levels inside the testing room environment to grasp the loudness level of its internal sound field; the measured sound pressure levels are plotted as shown in Figure 1. During testing, the tester held the BSWA-801 sound level meter 1.5 meters above the ground, conducting tests at each point for 3 min and repeating the measurements every 10 min, reading the sound pressure level values as displayed on the sound level meter (which shows the sound pressure level values to one decimal place). The recorded sound pressure level results are the average of the readings at each point. Figure 2 shows the curve of sound pressure level variations over time in the sound field (taking the average sound pressure level every 30 min). On the first day of sound pressure level measurements, the average sound pressure level was highest from 14:30 to 15:00, at 71.8 dB(A), and lowest from 15:00 to 15:30, at 64.0 dB(A). On the second day, the highest average was from 10:30 to 11:00, at 74.1 dB(A), and the lowest was from 15:00 to 15:30, at 65.3 dB(A). The average sound pressure level in the morning was 70.8 dB(A) and in the afternoon was 68.9 dB(A). This indicates that time variations have little impact on the sound pressure levels inside the pediatric dental clinic.

Next, the field questionnaire study was conducted. A stratified sampling method was used to divide the users of the pediatric dentistry clinic into two categories, i.e., healthcare workers and young pediatric patients. Respondents were then randomly selected, and it was confirmed that they had normal hearing before they were allowed to fill out the questionnaire. The survey questionnaire was based on the results of interviews and employed Method A from IOS (2018) for data collection. The questionnaire was compiled in Chinese and included sections on respondents’ basic information, overall sound environment assessment, respondents’ emotional perception, the impact of dominant sound sources on respondents’ emotions, and the sound sources they wished or did not wish to hear. Specific contents of the survey questionnaire are shown in Table 2. While the respondents were filling out the questionnaire, the BSWA-801 sound level meter was placed at the ear level of the respondents to measure the sound pressure level at their location within the sound field. Subsequently, 92 valid questionnaires were collected from the field study (30 were collected from healthcare workers, and 62 were collected from young pediatric patients); the number of valid questionnaires met the sample size requirement for applied statistics (Cheng and Hong, 2013).

**Table 2:** Research questionnaire content framework.

Questionnaire content		Options and quantitative information	
Background information (healthcare workers)	Gender	1: Male 2: Female	
	Age	1–25 ~ 35 years old 2–35 ~ 45 years old 3–45 ~ 55 years old 4->55 years old	
	Education level	1: Undergraduate 2: Graduate	
	Occupation	1: Doctor 2: Nurse	
	Years of work	1–1 ~ 3 years 2–3 ~ 5 years 3–5 ~ 8 years 4–8 ~ 10 years 5- > 10 years	
Background information (pediatric patients)	Gender	1: Male 2: Female	
	Age	1- < 3 years old 2–3 ~ 6 years old 3–6 ~ 11 years old	
	Dwell time	1- < 30 min 2–0.5 ~ 1 h 3–1 ~ 2 h 4- > 2 h	
Acoustic comfort evaluation (IOS Acoustic Standard Scale)		1-Very uncomfortable 2-Uncomfortable 3-Generally comfortable 4-Comfortable 5-Very comfortable	
Emotional perception evaluation (POMS emotional state scale)		1-Almost no impact 2-Low impact 3-Medium impact 4-High impact 5-Very high impact	
Evaluation of the influence of sound source on emotion		Type of sound source	Dental-type sound source (dental drill, saliva ejector, air/water syringe)
			Non-dental sound sources (conversations, children crying, parents crying/scolding)
		Degree of influence	1-Almost no impact 2-Low impact 3-Medium impact 4-High impact 5-Very high impact
The emotions and emotional responses of users within the soundscape of the clinic (Please check ‘√’ the item of your choice)		Positive emotions	□Pleasure □Relaxation
		Negative emotions	□Nervousness □Restlessness □Anxiety □Anger □Fear □Pain
		Emotional response	□Hostility
Sound source (dental and non-dental sound sources) Type of emotion (nervousness, restlessness, anxiety, anger, fear, and pain) Emotional response (hostility)		Taking nervousness as an example: 1-Not nervous at all 2-Not nervous 3-General nervousness 4-Nervous 5-Very nervous	

The results of this study were analyzed using the SPSS Statistics software (Qiu, 2013). Firstly, by using Pearson correlation analysis and regression analysis, it is determined that emotional perception is a factor influencing users’ soundscape evaluation in the pediatric dental clinic. Then, principal component analysis was conducted to determine the dominant sound sources affecting users’ emotional changes in the clinic. Pearson correlation analysis was implemented to determine the effects of dominant sound sources on users’ different emotional perception. Finally, an independent sample *t*-test and one-way ANOVA were used to determine the factors influencing other types of emotional perception brought on by the effect of dominant sound sources from demographic and social factor perspectives.

## 3 Results

### 3.1 Acoustic comfort assessment and emotional perception evaluation

During normal working hours, the background sound pressure level in the pediatric dentistry clinic fluctuated from 64 dB–79 dB, with a mean value of 69.9 dB (*SD* = 3.81); users’ assessments of the internal acoustic comfort were mainly rated as average (58.3%) and uncomfortable (38.3%), with very few ratings of very uncomfortable (1.7%) and comfortable (1.7%). By conducting a Pearson correlation analysis between the background sound pressure level and users’ acoustic comfort evaluations in the pediatric dental clinic, it was revealed that there is a significant positive correlation between users’ evaluations of acoustic comfort in the pediatric dental clinic and the background sound pressure level (*R* = 0.294, *p* < 0.05) (Qiu, 2013). The regression analysis results on the acoustic comfort evaluations and background sound pressure levels in the pediatric dental clinic show that the background sound pressure levels account for only 8.6% of the variance in users’ acoustic comfort evaluations (*R*^2^ = 0.086, *p* = 0.23 < 0.05). This may be due to users’ acoustic comfort evaluations improving as the background sound pressure levels increase within a certain threshold range, showing a highly positive correlation. However, beyond this threshold, users’ evaluations of the sound environment decrease (Chen and Kang, 2016). The Pearson correlation and regression analyses results indicated that background sound pressure levels did not fully explain the changes in users’ acoustic comfort evaluations in the pediatric dentistry clinic. This was consistent with the results of Yang and Kang (2005) in an urban public space soundscape study and those described by Kang et al. (2012) in an underground commercial street soundscape study.

**Figure 3:**
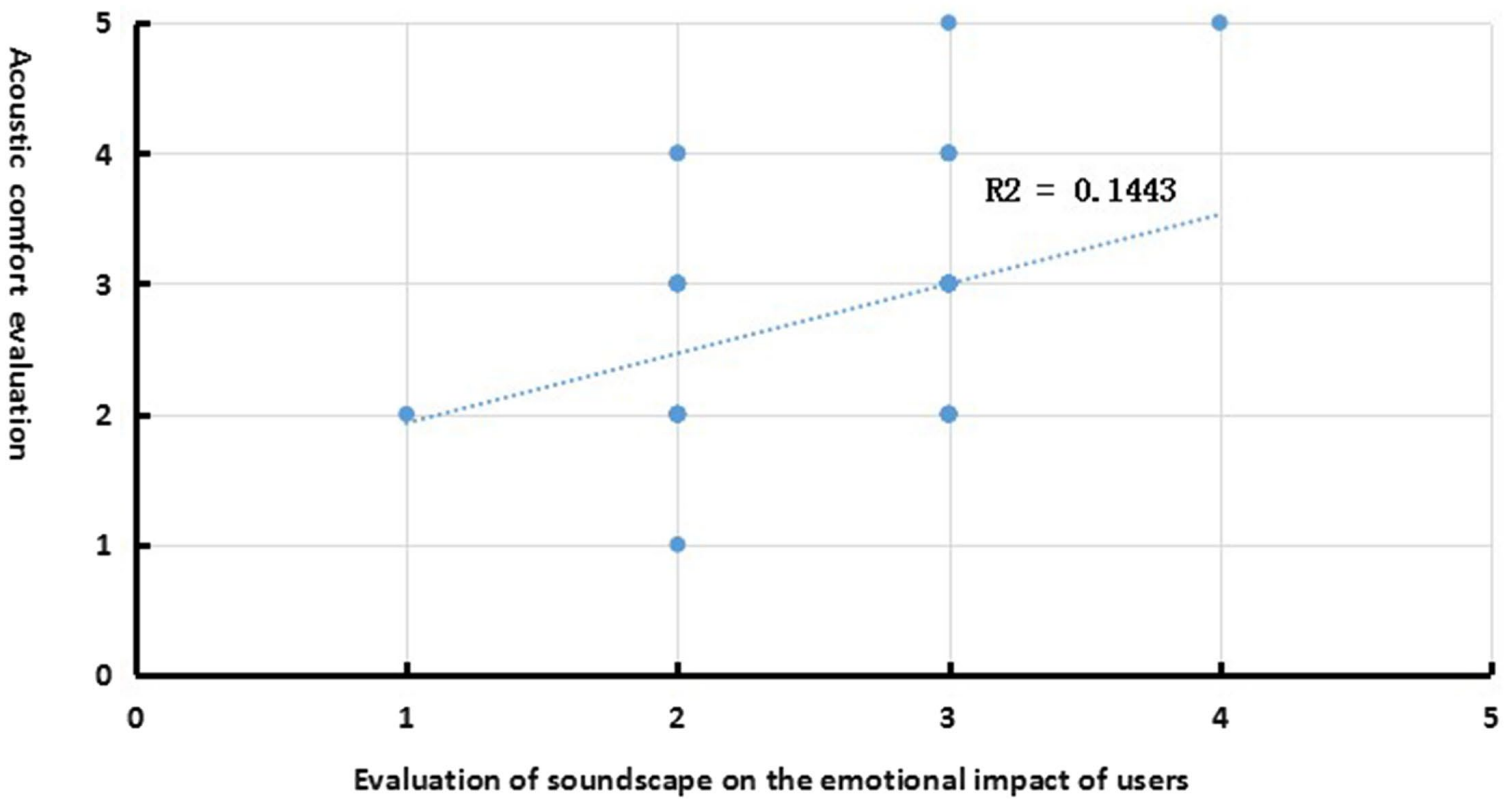
Linear regression of sound comfort evaluation and emotional impact evaluation.

The statistical results showed that 93.7% of the users felt that the acoustic comfort inside the pediatric dentistry clinic had an impact on their emotions, with the majority of users perceiving medium (50%) and high (35%) impacts and only a small number of users perceiving a low (10%) impact. The results of the Pearson correlation analysis between the users’ acoustic comfort ratings and the assessment of the soundscape’s perception on users’ emotions show a significant positive correlation in the pediatric dental clinic (*R* = 0.38, *p* < 0.05). Figure 3 shows the regression curve between the users’ acoustic comfort ratings and the assessment of the soundscape’s perception on users’ emotions. The assessment of emotional perception by users explains 14.43% of the variance in their acoustic comfort ratings (*R*^2^ = 0.1443, *p* = 0.036 < 0.05). The results indicate that the degree to which emotions are affected is a significant factor influencing users’ acoustic comfort ratings in pediatric dental clinics for young pediatric patients.

### 3.2 The effect of each independent sound source on the evaluation of emotional perception

**Figure 4:**
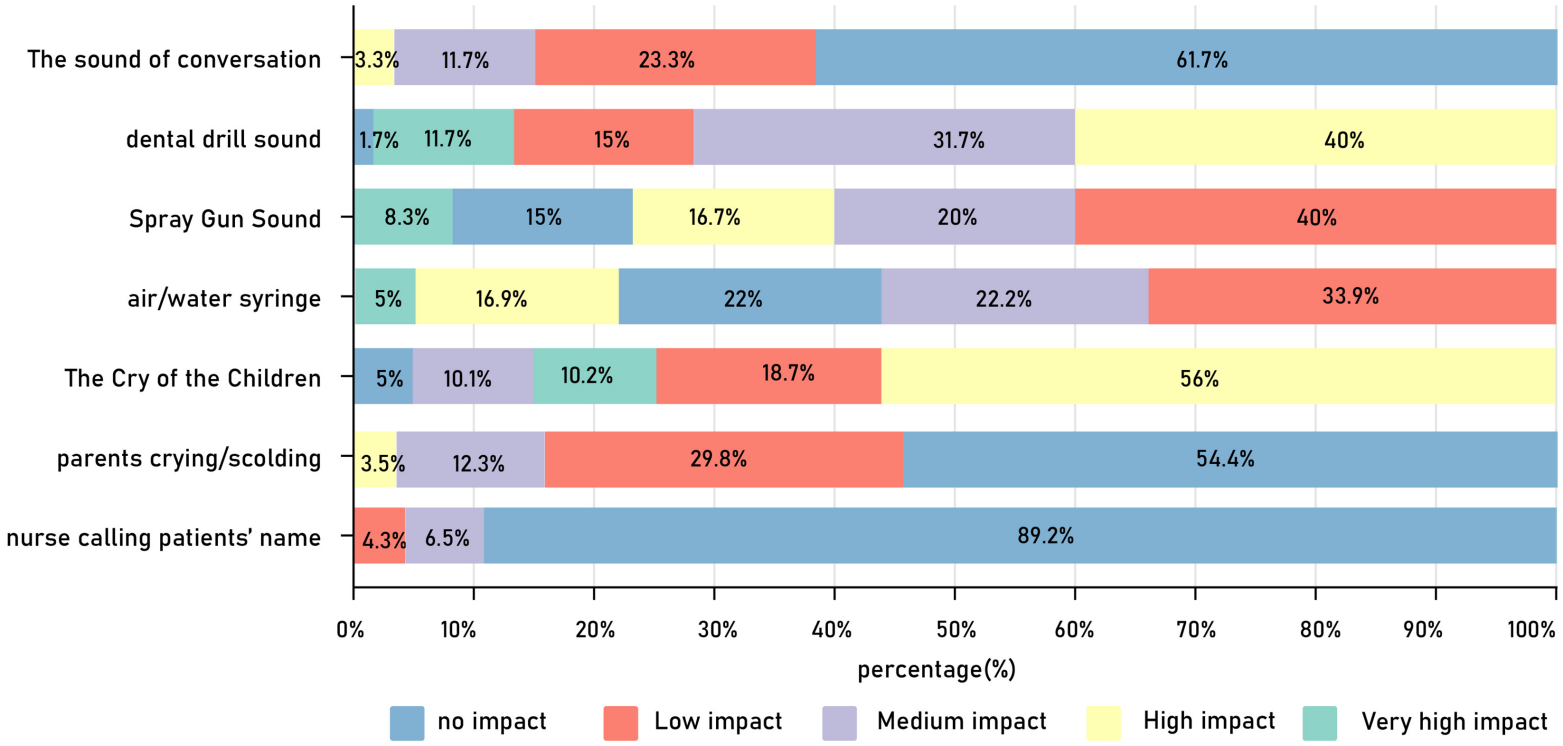
Distribution chart of percentage evaluation of the influence of each independent sound source on user emotions.

The statistics of each independent sound source within the background sound of the dental clinic for young pediatric patients (in the sound source identification, if the sound source is not heard by 80% of the interviewees, the sound source is statistically ignored). It can be observed that the dental sound sources perceived by users include sounds from dental drills, air/water syringes, and saliva ejectors. Non-dental sound sources included conversations, children crying, parents crying/scolding and nurse calling patients’ name. Figure 4 shows the statistical results of the evaluation of the emotional impact on users of each independent sound source within the background sound of the pediatric dental clinic. Among the non-dental sound sources, the sound of a child crying had a high impact on the users’ emotions; 95% of users thought that this sound impacted them emotionally, 10.2% of whom believed it had a very high impact, 56% denoted a high impact, 10.1% a medium impact and 18.7% of users thought it had an impact but to a lesser extent. In interviews, healthcare workers indicated that the sound of children crying triggers feelings of anxiety and restlessness; young pediatric patients reported that hearing other young pediatric patients crying causes them to experience fear, pain, and nervousness. Users were also receptive to speech-based non-dental sound sources; 89.2% thought that the sound of a nurse calling patients did not affect their emotions, and a small number of users thought it had a medium (6.5%) or low (4.3%) impact; 84.2% of users believed that the sound of parents talking had a low (29.8%) or almost no impact (54.4%) on their emotions, and only 3.3% of users felt that the sound of talking affected their emotions. This may have been because speech-based sound sources were a sound type that users anticipated hearing in a pediatric dentistry clinic (Liu et al., 2014).

Dental sound sources had an impact on the emotions of young pediatric patients, where 98.4% stated that the dental drill sound affected them emotionally; among them, 40% thought it had a high impact and 11.7% believed it had a very high impact. Additionally, 78% of the young pediatric patients feel that the sound of the saliva ejector affects their emotions, with 5% thought it had a very high impact. Among the young children, 25% thought that the air/water syringe sound had a high (16.7%) or even a very high (8.3%) impact on their emotions; however, the majority (50%) of these patients thought that this sound had a negligible impact on their emotions. These results were consistent with those of the questionnaire, which asked patients to list the sound sources that caused crying; here, the sound of dental drills (36 times), saliva ejectors (7 times), and air-water syringes (4 times) were mentioned at a high frequency.

Using the emotional perception of each independent sound source in the pediatric dental clinic as variables, and the evaluation of the emotional perception of the soundscape (very high, high, and medium emotional impacts) as selection variables for factor analysis. The results of the KMO and Bartlett’s test showed a KMO value of 0.617 (*p* < 0.001), and the results of the factor analysis showed that the selected variables were suitable for factor analysis (Qiu, 2013). The results of the factor analysis indicate that the sounds of the dental drill, air/water syringe, saliva ejector, and children crying are the main factors that can explain 87.041% of the variation in emotional perception evaluations by users in the pediatric dentistry clinic soundscape. The impact of these sound sources on user emotions, in order of strongest to weakest, is as follows: air/water syringe (39.505%), dental drill (22.061%), saliva ejector (13.465%), children crying (12.011%). Therefore, it can be concluded that the sounds of the dental drill, three-way syringe, saliva ejector, and children crying are the dominant sound sources affecting user emotions in pediatric dentistry clinic.

### 3.3 The effect of dominant sound sources on the evaluation of emotional perception

**Figure 5:**
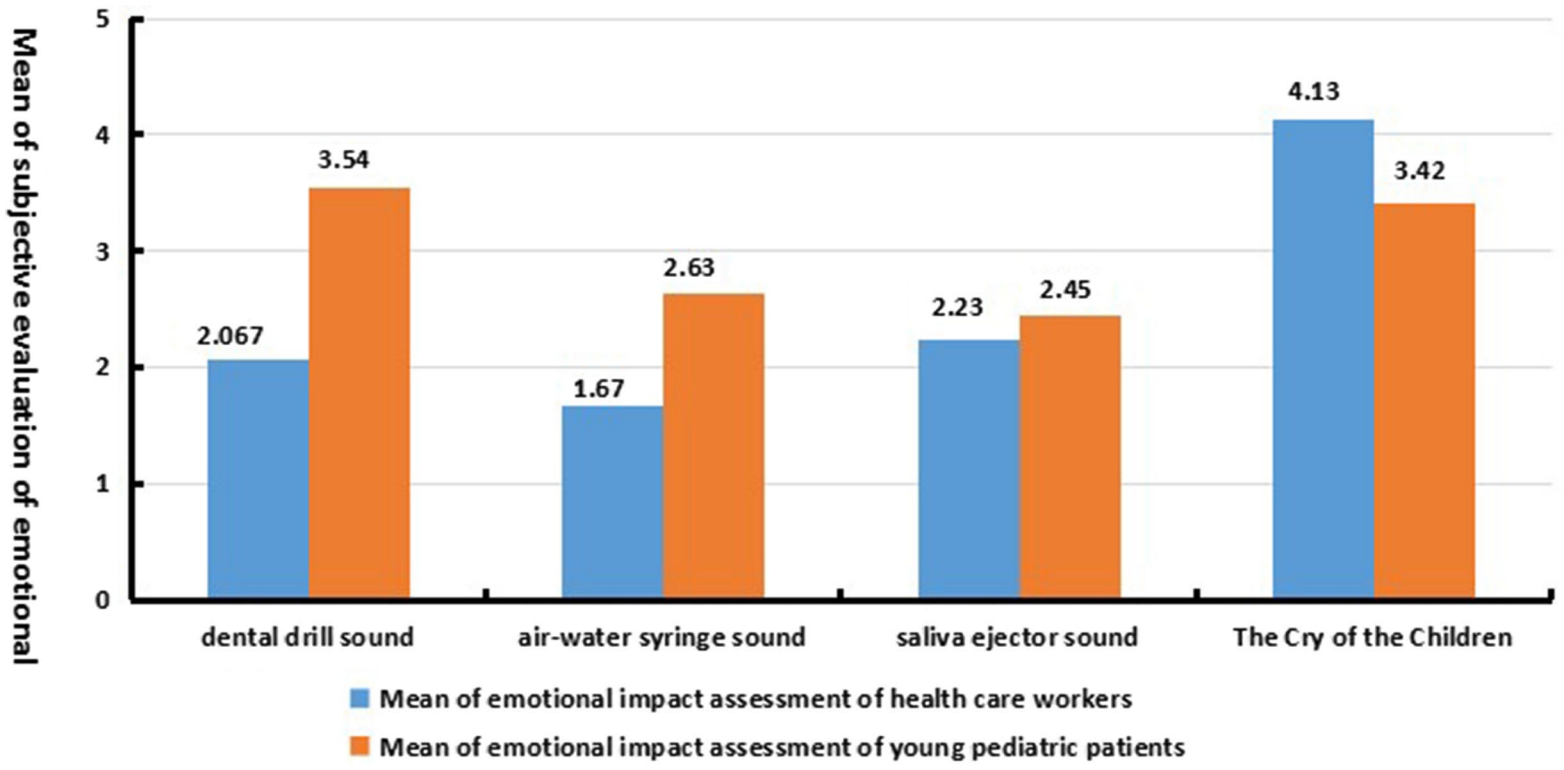
The dominant sound source affects the average value of user emotion perception impact evaluation.

The mean values of subjective evaluations of the influence of dominant sound sources on user emotions (as shown in Figure 5) showed that the healthcare workers’ emotions were weakly affected by primary dental category sound sources (dental drill, air/water syringe, and saliva ejector sounds). The mean values of subjective evaluations of the change in the degree of emotional influence ranged from 1.67 to 2.23, with standard deviations in the range of 1–1.22. However, the dominant sound sources in the non-dental category (children crying) had a more significant influence on healthcare workers’ emotions (*M* = 4.13, *SD* = 0.63). Compared to healthcare workers, dental sound sources have a stronger impact on the emotional states of young children patients; specifically, the average emotional perception rating of the dental drill sound on young children patients (*M* = 3.54, *SD* = 0.95) is 1.473 higher than that of healthcare workers, and the emotional perception rating of the air/water syringe sound (*M* = 2.63, *SD* = 1.18) is 0.96 higher than that of healthcare workers. The emotional perception of the saliva ejector noise on young children patients (*M* = 2.45, *SD* = 1.2) is only 0.12 higher than that on healthcare workers. However, non-dental dominant sound sources have a weaker emotional impact on young children patients (*M* = 3.42, *SD* = 1.15), being 0.71 lower than that on healthcare workers.

**Figure 6:**
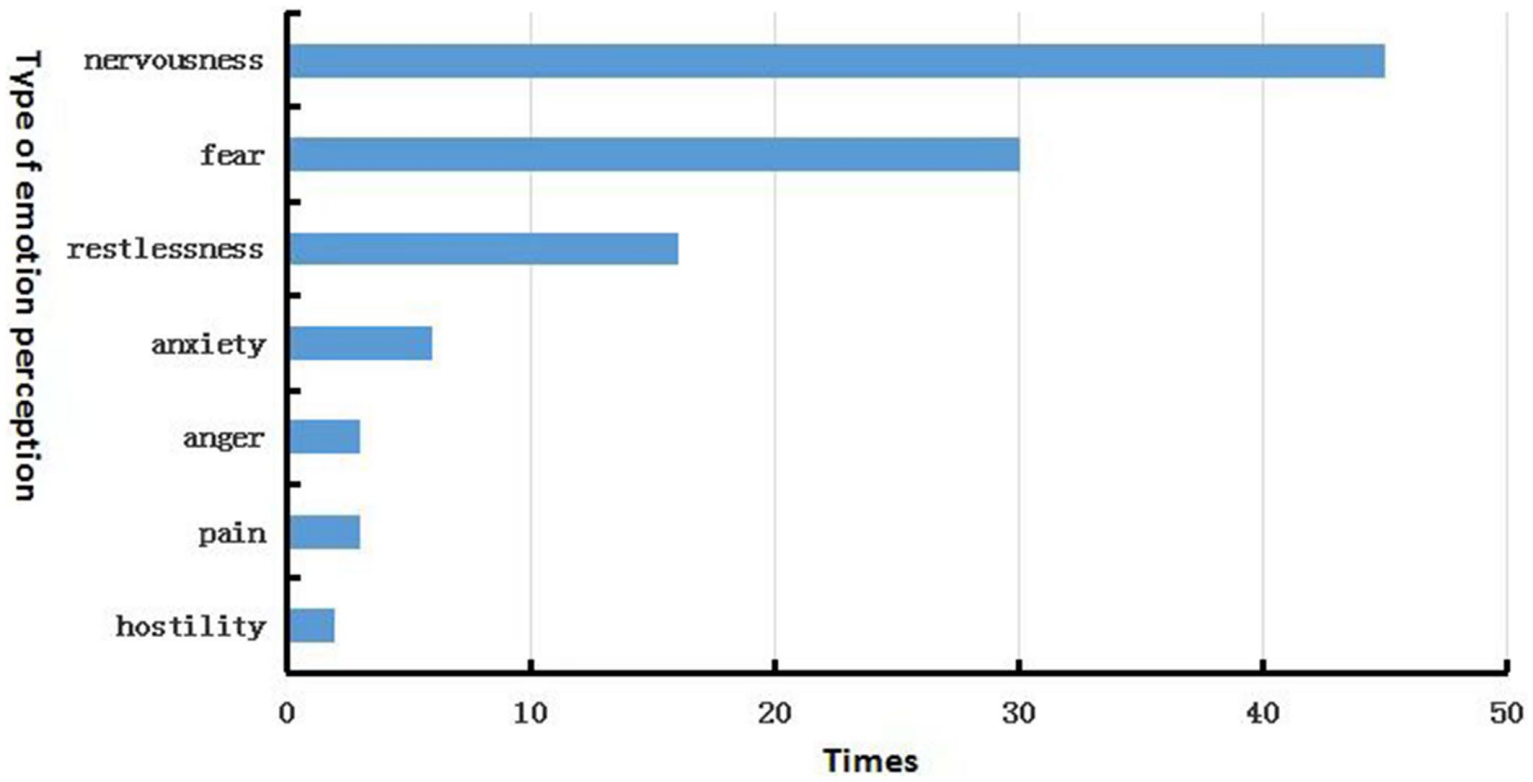
The statistical times of users perceiving different emotions in the sound environment.

The statistical results for the survey question ‘What impact does the sound in the clinic have on your emotions?’ (a multiple-choice question with options categorized into positive and negative emotions) are shown in Figure 6. Among these, nervousness was the most common emotion, and hostility the least. From this, we can conclude: it can be concluded that in the soundscape of pediatric dental clinics, the emotions of the users are primarily negative (no statistics on positive emotions are available). The predominant emotional perceptions are nervousness, fear, and restlessness, with a minority of users experiencing feelings of anxiety, anger, pain, and hostile emotional reactions. Among these, young pediatric patients exhibit perceptions of nervousness, restlessness, anger, fear, and hostility, while healthcare workers may experience feelings of nervousness, restlessness, anxiety, anger, fear, pain, and hostility.

By conducting bivariate correlation analysis between the subjective evaluation of various emotional perceptions of users under the influence of the dominant sound source and the subjective evaluation of the impact of the dominant sound source on users’ emotions, the results are shown in Table 3. For healthcare workers, the dominant dental sound source caused emotional experiences of nervousness, restlessness, anxiety, anger and pain, and there was a significant negative correlation (*p* < 0.05), indicates that under the influence of dental-related dominant sound sources, the stronger the emotional impact of the sound environment on healthcare workers, the weaker the perceptions of nervousness, restlessness, anxiety, anger and pain in their emotional responses.

**Table 3:** Subjective evaluation of the perception of distinct types of emotions and the subjective evaluation of the emotional impact of healthcare workers under the effect of dominant sound sources.

Type of sound source	Dominant sound source	Correlation test	Type of emotional perception					
			Nervousness	Restlessness	Anxiety	Anger	Fear	Pain
Dental sound sources	Dental drill sound	Pearson correlation	−0.442^*^	−0.451^*^	−0.577^**^	−0.396^*^		
		Sig. (2-tailed)	0.014	0.012	0.01	0.03		
	Air/water syringe sound	Pearson correlation	−0.443^*^		−0.338^*^		0.399^*^	
		Sig. (2-tailed)	0.017		0.034		0.029	
	Saliva ejector sound	Pearson correlation	−0.446^*^	−0.625^**^	−0.706^**^	−0.508^**^		−0.453^*^
		Sig. (2-tailed)	0.013	0.000	0.000	0.000		0.012
Non-dental sound sources	Children crying	Pearson correlation			0.317^*^			
		Sig. (2-tailed)			0.038			

^**^p ≤ 0.01, ^*^p ≤ 0.05.

This may have been due to long-term exposure to the same soundscape, where healthcare workers already had a tolerance to the sounds of the dental instruments and equipment (Phun et al., 2016). The degree of emotional perception analysis among healthcare workers under the effect of dental drill sounds showed a general negative correlation with the evaluation of the degree of nervousness (*R* = −0.442, *p* < 0.05), restlessness (*R* = −0.451, *p* < 0.05) and anxiety (*R* = −0.577, *p* < 0.01), as well as a relatively weak negative correlation with the evaluation of the degree of anger (*R* = −0.396, *p* < 0.05).

Under the effect of the saliva ejector sound, the degree to which healthcare workers’ emotions are affected shows a significant negative correlation with the development of feelings of nervousness (*R* = −0.446, *p* < 0.05), restlessness (*R* = −0.625, *p* < 0.01), anxiety (*R* = −0.706, *p* < 0.01), anger (*R* = −0.508, *p* < 0.01) and pain (*R* = −0.453, *p* < 0.05). Among these results, the degree of emotional impact on healthcare workers is very closely negatively correlated with the degrees of restlessness and anxiety evaluated, with correlation coefficients of −0.625 and −0.706, respectively, (*p* < 0.01). This indicates that the perception of the saliva ejector sound on their emotions is strong, and the greater this perception, the less pronounced are the feelings of restlessness and anxiety. This result indicated that the greater the degree of change in the mood of healthcare workers affected by the sound of the air/water syringe, the less significant the degree of restlessness and anxiety. There was a general negative correlation between the perceived emotional ratings for nervousness (*R* = −0.443, *p* < 0.05) and anxiety (*R* = −0.338, *p* < 0.05) among healthcare workers under the effect of the air/water syringe sound and the degree to which it impacted them emotionally. However, the perception of fear (*R* = 0.399, *p* < 0.05) among healthcare workers was positively correlated with their degree of emotional perception; this indicated that the greater the degree of the emotional perception among healthcare workers under the effect of the air/water syringe sound, the stronger the perception of fear. In addition, under the influence of non-dental dominant sound sources such as the crying of children, there is a significant presence of anxiety among healthcare workers (*R* = 0.317, *p* < 0.05).

Under the influence of dominant sound sources, there are significant differences in emotional perception between young pediatric patients and healthcare workers. As shown in Table 4, under the effect of dental-type sound sources, young pediatric patients experienced significant feelings of nervousness, restlessness, anger, fear, along with significant hostile reactions. Under the effect of the dental drill sound, the degree to which young pediatric patients’ emotions are affected is strongly correlated positively correlated with their perceived levels of nervousness (*R* = 0.501, *p* < 0.01), restlessness (*R* = 0.392, *p* < 0.05), fear (*R* = 0.428, *p* < 0.01) and the degree of hostile reactions (*R* = 0.261, *p* < 0.05). Among these, the extent of emotional impact on young pediatric patients is closely positively correlated with their levels of nervousness and fear, with correlation coefficients of 0.501 and 0.428, respectively, (*p* < 0.01), indicating that the greater the influence of the dental drill sound on the emotions of young pediatric patients, the stronger the sense of nervousness and fear they experienced. The degree to which young pediatric patients’ emotions are affected shows a lower correlation with the levels of perceived restlessness and the degree of hostile reactions, with the correlation to restlessness being higher than to hostile reactions, with correlation coefficients of 0.392 and 0.261, respectively, (*p* < 0.05). This indicates that the sound of the dental drill has a strong impact on the emotions of young pediatric patients, leading to a certain degree of restlessness and hostile reactions in their emotional changes. Under the influence of the air/water syringe sound and saliva ejector sound, the degree to which emotions are affected is closely positively correlated with the degree of hostile reactions ([*R*_ir-water syringe sound_ = 0.405, *p* < 0.01), (*R*_saliva ejector sound_ = 0.417, *p* < 0.01]); this indicates that under the influence of the air/water syringe and saliva ejector sounds, the emotional impact on young pediatric patients is strong, resulting in more stronger hostile reactions. Under the effect of an air/water syringe, the degree to which young pediatric patients’ emotions are affected shows a moderate correlation with their perceived levels of nervousness, anger (*R* = 0.380, *p* < 0.01) and restlessness (*R* = 0.367, *p* < 0.01). The perception of the saliva ejector sound on the emotional state of young pediatric patients shows a stronger correlation with their perceived levels of fear (*R* = 0.361, *p* < 0.01) and anger (*R* = 0.355, *p* < 0.01) compared to nervousness (*R* = 0.290, *p* < 0.05). Dental sound sources do not significantly affect the emotional perception of anxiety and pain in young pediatric patients (*p* > 0.05). Non-dental dominant sound sources (such as the sound of children crying) do not have a significant correlation with the emotional perception evaluation of young pediatric patients (*p* > 0.05).

**Table 4:** Subjective evaluation of diverse types of emotional perception and subjective evaluation of emotional impact in young pediatric patients under the effect of dominant sound sources.

Type of sound source	Dominant sound source	Correlation test	Type of emotional perception						
			Nervousness	Restlessness	Anxiety	Anger	Fear	Pain	Hostility
Dental sound sources	Dental drill sound	Pearson correlation	0.501^**^	0.392^*^			0.428^**^		0.261^*^
		Sig. (2-tailed)	0.000	0.002			0.001		0.044
	Air/water syringe sound	Pearson correlation		0.367^**^		0.380^**^	0.282^*^		0.405^**^
		Sig. (2-tailed)		0.004		0.003	0.029		0.001
	Saliva ejector sound	Pearson correlation	0.290^*^			0.355^**^	0.361^**^		0.417+
		Sig. (2-tailed)	0.025			0.005	0.005		0.001
Non-dental sound sources	Children crying	Pearson correlation							
		Sig. (2-tailed)							

^**^p ≤ 0.01, ^*^p ≤ 0.05.

**Figure 7:**
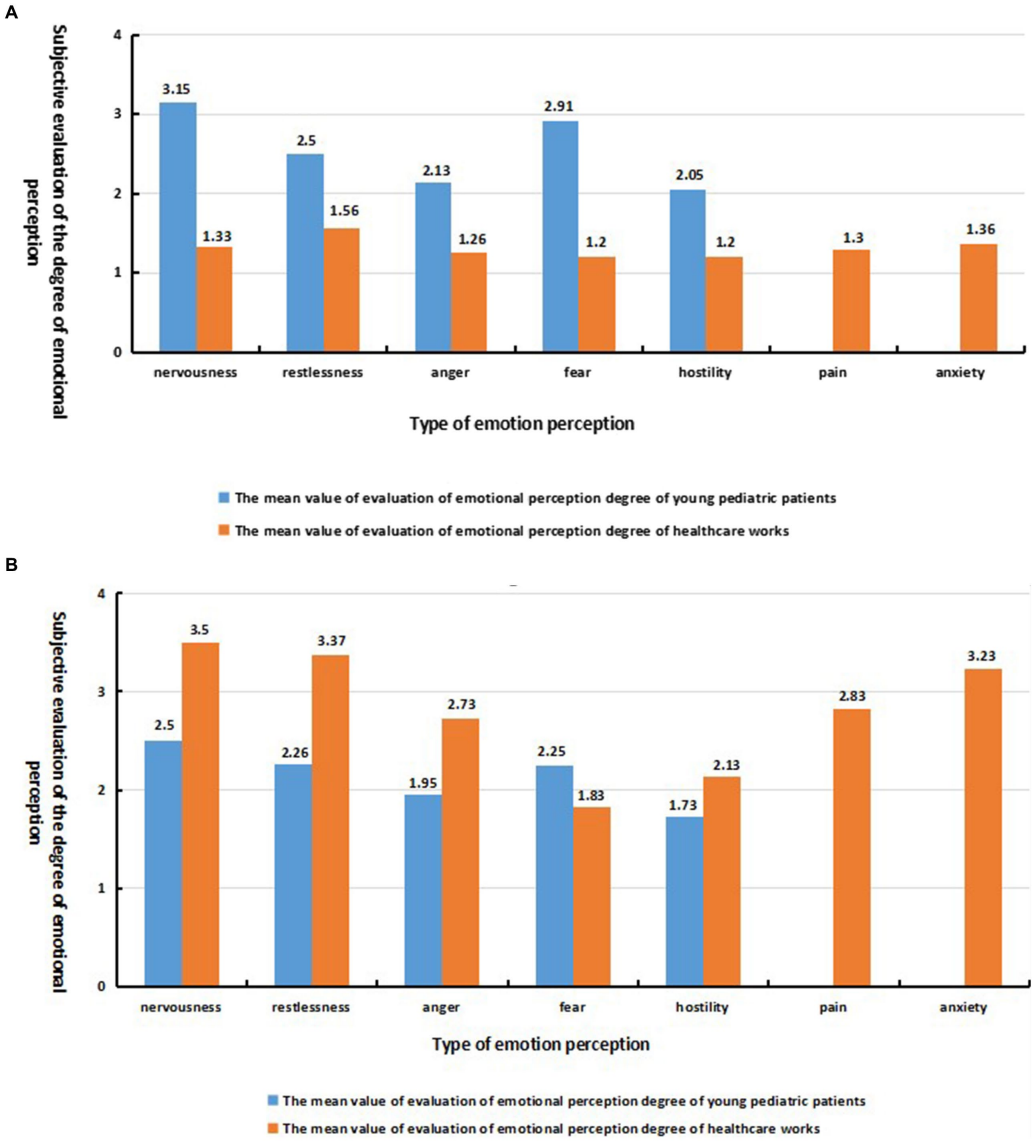
**(A)** Average perceptual evaluation of user emotions under dental sound source effect; **(B)** Average perceptual evaluation of user emotions under child crying sound source effect.

Figure 7A shows the mean values of users’ ratings for the degree of experienced emotion in each category under the effect of the dominant sound source. The mean values of negative emotion ratings among healthcare workers, influenced by the dominant sound source in the dental category, ranged from 1.2 to 1.56, which indicated a smooth emotional experience and a weak degree of negative emotion perception. However, young children evidenced a strong sense of nervousness (*M* = 3.15, *SD* = 1.07) and fear (*M* = 2.91, *SD* = 1.18). The mean evaluation value of the perceived degree of nervousness was 1.82 higher than that of healthcare workers, and the mean evaluation value of the perceived degree of fear was 1.71 higher compared with healthcare workers. This is reflected in what pediatric patients stated in the interview:

“The sound of the dental drill will make me feel afraid and also make me feel pain.”

“I get nervous and afraid of pain when it’s my turn to see a dentist.”

“I do not like to hear the sound of [the] air/water syringe, and I hope to hear the soothing sound of my mother or the sound of nursery songs.”

Under the influence of the non-dental dominant sound source, the emotional evaluation of the young pediatric patients was more stable compared with the healthcare workers (as shown in Figure 7B), but a slight sense of nervousness (*M* = 2.5, *SD* = 1.37) and fear (*M* = 2.25, *SD* = 1.2) was observed. This is evidenced by the pediatric patients in the interviews:

“I also feel pain when I hear crying.”

“I am afraid of pain.”

“I am afraid and nervous when I go to the dentist.”

perception of fearfulness were weaker among the healthcare workers (*M* = 1.2, *SD* = 0.55), but feelings of nervousness (*M* = 3.5, *SD* = 1.04), restlessness (*M* = 3.37, *SD* = 0.93) and anxiety (*M* = 3.23, *SD* = 1.22) were more potent, and a mild sense of pain (*M* = 2.83, *SD* = 0.99) was observed at the sound of a child crying. This is highlighted by the healthcare workers in the interviews:

“Working in this noisy environment for a long time can lead to hearing loss, and also a sense of depression and pressure,”

“Patients crying during treatment makes me a little overwhelmed.”

“I am used to the sound of work equipment, but the sound of young pediatric patients crying makes me [feel] anxious, nervous and helpless.”

### 3.4 The influence of population/social factors on emotional perception

Individual and group sensitivity to sound reflects attitudes and perception of environmental noise, and demographic and social factors play an important role in evaluating the soundscape (Kang, 2011). In this study, users in the pediatric dentistry clinic were divided into two categories, i.e., healthcare workers and young pediatric patients. An independent samples *t*-test and one-way ANOVA were conducted to determine the influencing factors that caused significant emotional experiences (nervousness, restlessness, anxiety, anger, pain, and fear) and emotional responses (hostility) in users under the effect of dominant sound sources, based on demographic and social factors.

In the healthcare workers’ sample, there are 18 doctors and 12 nurses. 80% of the healthcare workers are female, and 82.3% are under the age of 45, with 60% aged between 25 and 35 years, 22.3% aged between 35 and 45 years, and only 4.4% in the 45 to 55 age bracket; classified by educational level, 56.7% of the healthcare workers have a postgraduate degree, while 43.3% have an undergraduate degree.

In the young pediatric patients’ sample, male and female patients each account for 50%, with the majority being young children aged 4 to 11 years (91.7%). 73.3% of these patients spend less than 30 min in the examination room, while 8.3% stay for more than an hour.

#### 3.4.1 Healthcare workers

##### 3.4.1.1 Age

The age factor significantly affected the perceived evaluation of fear in healthcare workers (*p* = 0.029, *p* < 0.05). Under the effect of the dominant sound source, the majority (93.3%) of healthcare workers do not experience strong fear (86.7% are not scared at all, and 6.7% are not scared), with only 6.7% feeling slightly fearful (choosing ‘moderate’ in the survey options). The results of the one-way ANOVA showed that the level of fear was strongest among healthcare workers aged 45–55 years (*M* = 3.0), while those aged 25–35 (*M* = 2.0) and 35–45 years (*M* = 1.5) have weaker or no feelings of fear.

##### 3.4.1.2 Occupation

Occupational factors significantly affected the perceived evaluation of fear among healthcare workers (*p* = 0.02 < 0.05). The results of the independent samples t-test showed that there was a significant difference in the experience of fear between doctors and nurses. The perceived level of fear among nurses (*M* = 2.33, *SD* = 1.07) was 0.83 stronger than for physicians (*M* = 1.5, *SD* = 0.79).

##### 3.4.1.3 Years of work experience

Years of work experience showed a significant difference in the evaluation of the restlessness experienced by healthcare workers (*p* = 0.027 < 0.05). The majority (66.7%) of healthcare workers stated that, based on the dominant sound source, they were ‘not irritable at all’, 10% said they were ‘not irritable’ but 23.3% indicated they experienced restlessness. The results of one-way ANOVA showed that the restlessness of healthcare workers with 8–10 years of experience was the strongest (*M* = 4.31, *SD* = 0.22); this was 0.87 stronger than the restlessness of workers with 3–5 years of experience, and the restlessness of workers with 1–3 years of experience (*M* = 3.33, *SD* = 1.045) was minimally lower (0.11) than the mean for 3–5 years of experience. Healthcare workers with more than 10 years of work experience experienced less restlessness (*M* = 3, *SD* = 0.21).

The effect of gender and education level on the emotional evaluation of healthcare workers in the pediatric dentistry clinic was not significant (*p* > 0.05) under the effect of the dominant sound source.

#### 3.4.2 Young pediatric patients

Age was an important factor that gave rise to a significant difference in the evaluation of hostility among young pediatric patients (*p* = 0.016, *p* < 0.05). Under the influence of the dominant sound source, a majority (58.3%) of young children patients exhibited noticeable hostile reactions, with 10% of the young children patients showing strong hostile reactions. The results of the one-way ANOVA showed that, based on the age group of pediatric dental patients, infant patients (<3 years old) reflected the strongest sense of hostility (*M* = 2.6, *SD* = 0.89), children (7–11 years old) generated hostile reactions at a level only slightly lower (0.37) than infant patients, and preschool-aged children (4–6 years old) showed the weakest hostile reactions (*M* = 1.85, *SD* = 0.92).

Gender and dwell time factors did not have a significant effect (*p* > 0.05) on the emotional experience evaluation of young pediatric patients under the effect of the dominant sound source.

## 4 Conclusion

This article selects a typical pediatric dental clinic as the research subject, using a combination of on-site subjective questionnaires and objective tests. It investigates the correlation between the dominant sound source and the emotions of users during the stable periods of the sound field in the pediatric dental clinic. The results indicate that the background sound pressure level inside the pediatric dental clinic ranges from 64 to 79 dB, with most users (96.6%) considering the soundscape to be acceptable but not comfortable. There is a significant positive correlation between users’ evaluations of sound comfort in the clinic and the background sound pressure level (*p* < 0.05), although the background sound pressure level does not fully explain users’ perceptions of sound comfort. The degree to which emotions are affected is another factor influencing users’ evaluations of sound comfort; in the clinic’s soundscape, users’ emotions are negative. Healthcare workers experience perceptions of nervousness, restlessness, anxiety, anger, fear, pain, and hostile emotional responses, while young pediatric patients exhibit perceptions of nervousness, restlessness, anger, fear, and hostile emotional responses.

In the clinic’s soundscape, the dominant sound sources that significantly affect users’ emotional changes can be divided into dental sound sources (dental drill sound, air-water syringe sound, saliva ejector sound) and non-dental sound sources (children crying). Under the influence of dental dominant sound sources, healthcare workers are less affected emotionally compared to young pediatric patients, and there is a significant negative correlation between the extent of healthcare workers’ emotional perception and their negative emotions (*p* < 0.05). Conversely, there is a significant positive correlation between the emotional impact on young pediatric patients and their negative emotions (*p* < 0.05). Young pediatric patients experience a significantly stronger perception of nervousness, being 1.82 higher, and fear, being 1.71 higher, compared to healthcare workers. Under the influence of non-dental dominant sound sources, healthcare workers are more strongly affected emotionally compared to young pediatric patients, with a significant increase in anxiety as the level of emotional perception strongly (*p* < 0.05). Healthcare workers’ perceptions of nervousness and restlessness are, respectively, 1 and 1.11 higher than those of young pediatric patients, and also feel mild pain; however, there is no significant correlation between the emotional perception on young pediatric patients and their emotional perception within the soundscape (*p* > 0.05).

In terms of demographic/social factors, age, profession, and working years factor significantly influence the emotional perception of healthcare workers (*p* < 0.05); healthcare workers aged 45–55 experience the strongest sense of restlessness, and nurses have a fear perception that is 0.83 times stronger than that of doctors. Age also significantly affects the hostile responses of young pediatric patients (*p* < 0.05), with toddlers (under 3 years old) showing a 0.37 times stronger hostile response compared to patients aged 4–11 years.

Based on the research findings, it can be concluded that soundscapes are crucial for creating a comfortable dental environment for young children. To achieve this, the design of the dental clinic’s soundscape should start by clearly dividing the treatment area from the waiting area and soundproofing them to prevent noise from the waiting area from raising the sound pressure levels in the treatment area or affecting the emotions of medical staff and young patients due to children crying. However, sound pressure levels alone do not represent the perceived quality of sound (Torresin et al., 2019a); reducing noise levels does not necessarily increase comfort, and loudness can sometimes be “desirable” (Aletta et al., 2017). Furthermore, eliminating unwanted sound sources while enhancing desired ones can optimize the internal soundscape by incorporating sound sources that are highly favored by patients (for example, in pediatric dental clinics, sounds from cartoons, children’s songs, and storytelling) (Torresin et al., 2019b). Additionally, indoor spaces may be affected by both external and internal sound sources. Therefore, a suitable combination of indoor and outdoor sound sources can be made based on perceptual needs (Torresin et al., 2020b); for instance, creating sounds of water outdoors as a sound mask (Puyana-Romero et al., 2021).

This study did not conduct field tests on the sound field of pediatric dental clinics during non-working hours, nor did it explore the three perceptual dimensions of sound events—comfort, pleasantness, and familiarity—and their relationships with sound sources, loudness, and emotions (Torresin et al., 2020a,b). When researching the correlation between sound sources and the evaluation of acoustic comfort, the relationship between sound sources, their loudness, and emotions was not considered. These omissions limit the findings of the study. Future research will address these shortcomings, overcome limitations, and propose more targeted methods and strategies for designing and creating soundscapes in dental clinics.

## Data availability statement

The datasets presented in this article are not readily available because if necessary, please contact the author to obtain. Requests to access the datasets should be directed to YL, 569155569@qq.com.

## Ethics statement

The studies involving humans were approved by School of Art and Design, Zhejiang Sci-Tech University. The studies were conducted in accordance with the local legislation and institutional requirements. Written informed consent for participation in this study was provided by the participants’ legal guardians/next of kin.

## Author contributions

YL: Writing – review & editing, Writing – original draft, Investigation, Formal analysis, Conceptualization. XC: Writing – review & editing, Investigation, Formal analysis, Data curation, Conceptualization.

## References

[ref1] Akbakhanzadeh F. (1978). Effects of high-speed drill noise on dentists’ hearing. Iran. J. Public Health 7, 168–179,

[ref2] Aletta F. Botteldooren D. Thomas P. Vander Mynsbrugge T. De Vriendt P. Van de Velde D.. (2017). Monitoring sound levels and soundscape quality in the living rooms of nursing homes: a case study in Flanders (Belgium). Appl. Sci. 7:874. doi: 10.3390/app7090874

[ref3] Altinöz H. C. Gökbudak R. Bayraktar A. (2001). A pilot study of measurement of the frequency of sounds emitted by high-speed dental air turbines. J. Oral Sci. 43, 189–192. doi: 10.2334/josnusd.43.189, PMID: 11732739

[ref4] Axelsson Ö. Nilsson M. E. Berglund B. (2010). A principal components model of soundscape perception. J. Acoust. Soc. Am. 128, 2836–2846. doi: 10.1121/1.3493436, PMID: 21110579

[ref5] Babisch W. (2003). Stress hormones in the research on cardiovascular effects of noise. Noise Health 5, 1–11, PMID: 12631430

[ref6] Bali N. Acharya S. Anup N. (2007). An assessment of the effect of sound produced in a dental clinic on the hearing of dentists. Oral Health Prev. Dent. 5, 187–191, PMID: 17977289

[ref7] Cain R. Jennings P. Poxon J. (2013). The development and application of the emotional dimensions of a soundscape. Appl. Acoust. 74, 232–239. doi: 10.1016/j.apacoust.2011.11.006

[ref8] Chen W.-L. Chen C.-J. Yeh C.-Y. Lin C.-T. Cheng H.-C. Chen R.-Y. (2013). Workplace noise exposure and its consequent annoyance to dentists. J. Exp. Clin. Med. 5, 177–180. doi: 10.1016/j.jecm.2013.08.009

[ref9] Chen X. Kang J. (2016). Research on sound level threshold of underground restaurant space based on sound comfort. Appl. Acoust. 35, 157–164,

[ref10] Cheng J. H. Hong W. (2013). Principles and applications of statistics. Beijing: People’s Post and Telecommunications Publishing House.

[ref11] Chopra S. Pandey S. (2007). Occupational hazards among dental surgeons. Med. J. Armed Forces India 63, 23–25. doi: 10.1016/S0377-1237(07)80100-6, PMID: 27407931 PMC4921682

[ref12] Fernandes J. C. Ovileira J. R. E. Fernandes V. M. (2012). Avaliação do ruído em consultório odontológico. XI SIMPEP. Bauru/SP. 2004. Int Arch Otorhinolaryngol São Paulo-Bras. 16, 226–231,

[ref13] Gijbels F. Jacobs R. Princen K. (2006). Potential occupational health problems for dentists in Flanders, Belgium. Clin. Oral Investig. 10, 8–16. doi: 10.1007/s00784-005-0003-6, PMID: 16177883

[ref14] Herranz-Pascual K. Aspuru I. Iraurgi I. Santander Á. Eguiguren J. L. García I. (2019). Going beyond quietness: determining the emotionally restorative effect of acoustic environments in urban open public spaces. Int. J. Environ. Res. Public Health 16:1284. doi: 10.3390/ijerph16071284, PMID: 30974811 PMC6479382

[ref15] Hyson J. M. (2002). The air turbine and hearing loss: are dentists at risk? J. Am. Dent. Assoc. 133, 1639–1642. doi: 10.14219/jada.archive.2002.0113, PMID: 12512663

[ref16] IOS (2018). ISO/TS 12913-2:2018 - acoustics — soundscape — part 2: data collection and reporting requirements. Int. Stand. Organ. Available at: https://www.iso.org/standard/75267.html (Accessed April 2, 2024).

[ref17] ISO (2014). Acoustics — Soundscape — Part 1: Definition and conceptual framework. ISO. Available at: https://www.iso.org/standard/52161.html (Accessed April 2, 2024).

[ref18] Jadid K. Klein U. Meinke D. (2011). Assessment of noise exposures in a pediatric dentistry residency clinic. Pediatr. Dent. 33, 342–347, 21903003

[ref19] Jo H. I. Jeon J. Y. (2021). Urban soundscape categorization based on individual recognition, perception, and assessment of sound environments. Landsc. Urban Plan. 216:104241. doi: 10.1016/j.landurbplan.2021.104241

[ref20] Jones D. M. Davies D. R. (1984). Individual and group differences in the response to noise. Noise Soc. 125–153,

[ref21] Kadanakuppe S. Bhat P. K. Jyothi C. (2011). Assessment of noise levels of the equipments used in the dental teaching institution, Bangalore. Indian J. Dent. Res. 22, 424–431. doi: 10.4103/0970-9290.87065, PMID: 22048583

[ref22] Kang J. (2011). Theory of urban sound environment. Beijing: Science Press.

[ref23] Kang J. Meng Q. Jin H. (2012). Effects of individual sound sources on the subjective loudness and acoustic comfort in underground shopping streets. Sci. Total Environ. 435-436, 80–89. doi: 10.1016/j.scitotenv.2012.06.105, PMID: 22846767

[ref24] Kjellberg A. Landström U. Tesarz M. Söderberg L. Akerlund E. (1996). The effects of non-physical noise characteristics, ongoing task and noise sensitivity on annoyance and distraction due to noise at work. J. Environ. Psychol. 16, 123–136. doi: 10.1006/jevp.1996.0010

[ref25] Kong P.-R. Han K.-T. (2024). Psychological and physiological effects of soundscapes: a systematic review. Sci. Total Environ. 929:172197. doi: 10.1016/j.scitotenv.2024.172197, PMID: 38582113

[ref26] Liu J. Kang J. Behm H. Luo T. (2014). Effects of landscape on soundscape perception: soundwalks in city parks. Landsc. Urban Plan. 123, 30–40. doi: 10.1016/j.landurbplan.2013.12.003

[ref27] Lopes A. C. de Melo A. D. P. Santos C. C. (2012). A study of the high-frequency hearing thresholds of dentistry professionals. Int. Arch. Otorhinolaryngol. 16, 226–231. doi: 10.7162/S1809-97772012000200012, PMID: 25991940 PMC4432522

[ref28] Lorenzino M. D’Agostin F. Rigutti S. Bovenzi M. Fantoni C. Bregant L. (2020). Acoustic comfort depends on the psychological state of the individual. Ergonomics 63, 1485–1501. doi: 10.1080/00140139.2020.1808249, PMID: 32780646

[ref29] Masullo M. Maffei L. Iachini T. Rapuano M. Cioffi F. Ruggiero G.. (2021). A questionnaire investigating the emotional salience of sounds. Appl. Acoust. 182:108281. doi: 10.1016/j.apacoust.2021.108281

[ref30] Messano G. A. Petti S. (2012). General dental practitioners and hearing impairment. J. Dent. 40, 821–828. doi: 10.1016/j.jdent.2012.06.006, PMID: 22750643

[ref31] Mojarad F. Massum T. Samavat H. (2009). Noise levels in dental offices and laboratories in Hamedan. Iran 6, 181–186,

[ref32] Muppa R. Bhupatiraju P. Duddu M. Penumatsa N. V. Dandempally A. Panthula P. (2013). Comparison of anxiety levels associated with noise in the dental clinic among children of age group 6-15 years. Noise Health 15, 190–193. doi: 10.4103/1463-1741.112371, PMID: 23689302

[ref33] Özcan E. (2014). The Harley effect: internal and external factors that facilitate positive experiences with product sounds. J. Sonic Stud. 6:a07,

[ref34] Özcan E. Broekmeulen C. L. H. Luck Z. A. van Velzen M. Stappers P. J. Edworthy J. R. (2022). Acoustic biotopes, listeners and sound-induced action: a case study of operating rooms. Int. J. Environ. Res. Public Health 19:16674. doi: 10.3390/ijerph192416674, PMID: 36554556 PMC9779544

[ref35] Özcan E. van Egmond R. (2012). Basic semantics of product sounds. Int. J. Des. 6:2,

[ref36] Phun V. K. Hirata T. Yai T. (2016). Effects of noise information provision on aircraft noise tolerability: results from an experimental study. J. Air Transp. Manag. 52, 1–10. doi: 10.1016/j.jairtraman.2015.11.005

[ref37] Porritt J. Buchanan H. Hall M. (2013). Assessing children’s dental anxiety: a systematic review of current measures. Community Dent. Oral Epidemiol. 41, 130–142. doi: 10.1111/j.1600-0528.2012.00740.x, PMID: 22970833

[ref38] Puyana-Romero V. Maffei L. Brambilla G. Nuñez-Solano D. (2021). Sound water masking to match a waterfront soundscape with the users’ expectations: the case study of the seafront in Naples, Italy. Sustain. For. 13:371. doi: 10.3390/su13010371

[ref39] Qin Y. Zhao W. Kang J. (2020). The influence of background sound source types in elderly facilities’ activity spaces on emotions and activities. J. West. Hum. Settl. Environ. 35, 43–49. doi: 10.13791/j.cnki.hsfwest.20200406

[ref40] Qiu H. (2013). Quantitative research and statistical analysis-analysis of SPSS. Chongqing: Chongqing University Press.

[ref41] Russell J. A. (1980). A circumplex model of affect. J. Pers. Soc. Psychol. 39, 1161–1178. doi: 10.1037/h0077714

[ref42] Russell J. A. (2003). Core affect and the psychological construction of emotion. Psychol. Rev. 110, 145–172. doi: 10.1037/0033-295X.110.1.145 12529060

[ref43] Singh S. Gambhir R. S. Singh G. Sharma S. Kaur A. (2012). Noise levels in a dental teaching institute-a matter of concern! J Clin Exp Dent 4:e141, –e145. doi: 10.4317/jced.50725 24558544 PMC3917637

[ref44] Torresin S. Albatici R. Aletta F. Babich F. Kang J. (2019a). Assessment methods and factors determining positive indoor soundscapes in residential buildings: a systematic review. Sustain. For. 11:5290. doi: 10.3390/su11195290

[ref45] Torresin S. Albatici R. Aletta F. Babich F. Oberman T. Kang J. (2019b). Acoustic design criteria in naturally ventilated residential buildings: new research perspectives by applying the indoor soundscape approach. Appl. Sci. 9:5401. doi: 10.3390/app9245401

[ref46] Torresin S. Albatici R. Aletta F. Babich F. Oberman T. Siboni S.. (2020a). Indoor soundscape assessment: a principal components model of acoustic perception in residential buildings. Build. Environ. 182:107152. doi: 10.1016/j.buildenv.2020.107152

[ref47] Torresin S. Aletta F. Babich F. Bourdeau E. Harvie-Clark J. Kang J.. (2020b). Acoustics for supportive and healthy buildings: emerging themes on indoor soundscape research. Sustain. For. 12:6054. doi: 10.3390/su12156054

[ref48] Wong H. M. Mak C. M. To W. M. (2015). Development of a dental anxiety provoking scale: a pilot study in Hong Kong. J. Dent. Sci. 10, 240–247. doi: 10.1016/j.jds.2014.09.003

[ref49] Wong H. M. Mak C. M. Xu Y. F. (2011). A four-part setting on examining the anxiety-provoking capacity of the sound of dental equipment. Noise Health 13, 385–391. doi: 10.4103/1463-1741.90291, PMID: 22122954

[ref50] Xu X. Zhuang A. Han F. (2019). The influence of Main tones on the emotional perception of natural protected area soundscapes—a case study of Wulingyuan world heritage site. Chin. Landsc. Archit. 35:6. doi: 10.19775/j.cla.2019.08.0028

[ref51] Yang W. Kang J. (2005). Acoustic comfort evaluation in urban open public spaces. Appl. Acoust. 66, 211–229. doi: 10.1016/j.apacoust.2004.07.011

[ref52] Yang M. Kang J. (2013). Psychoacoustical evaluation of natural and urban sounds in soundscapes. J. Acoust. Soc. Am. 134, 840–851. doi: 10.1121/1.4807800, PMID: 23862890

[ref53] Zhang B. Kang J. (2022). Effect of environmental contexts pertaining to different sound sources on the mood states. Build. Environ. 207:108456. doi: 10.1016/j.buildenv.2021.108456

